# Comparing Outcomes After Referral to Physical Therapy for Patients With Glenohumeral Osteoarthritis Based on the Radiographic Osteoarthritis Severity: A Retrospective Analysis

**DOI:** 10.7759/cureus.43027

**Published:** 2023-08-06

**Authors:** Anthony N Baumann, Thad Indermuhle, Caleb J Oleson, Megan E Callaghan, Hudson Rogers, Caroline Pennacchio, Deven P Curtis, John Martin Leland

**Affiliations:** 1 Department of Rehabilitation Services, University Hospitals, Cleveland, USA; 2 College of Medicine, Northeast Ohio Medical University, Rootstown, USA; 3 College of Medicine, Case Western Reserve University, Cleveland, USA; 4 Department of Orthopedic Surgery, University Hospitals, Cleveland, USA

**Keywords:** radiographic severity, rehabilitation, orthopedics, physical therapy, musculoskeletal shoulder pain, glenohumeral osteoarthritis

## Abstract

Introduction

Glenohumeral osteoarthritis (GHOA) is a common cause of musculoskeletal pain (MSP) that can frequently lead to pain and functional disability in patients throughout the world. GHOA can be managed with conservative or surgical interventions, although conservative interventions, such as physical therapy (PT), are generally first-line interventions depending on the severity of GHOA. The purpose of this retrospective analysis was to examine how conventional PT impacts outcomes for patients with GHOA based on the severity of radiographic GHOA findings.

Methods

This study is a retrospective chart review of patients who were referred to PT for MSP and received PT in the outpatient setting between 2016 and 2022. Inclusion criteria were patients who received PT in the outpatient setting, received PT for MSP, had shoulder radiograph imaging within two years of initial PT evaluation, had more than one PT visit (i.e. attended a follow-up session after initial evaluation), and did not have a history of shoulder surgery. Primary outcome measures were pain, abduction active range-of-motion (AROM), and disability via the quick disabilities of the arm, shoulder, and hand (DASH). Patients were divided into the No GHOA group (n=104), Mild GHOA group (n=61), and Moderate/Severe GHOA group (n=55) based on the radiographic GHOA severity.

Results

All included patients (n=220) had a mean age of 62.2 ± 12.4 years old with a mean number of PT visits of 7.8 ± 4.5 visits. There was initially a significant difference in the magnitude of pain improvement between the three groups based on radiographic severity of GHOA (Kruskal-Wallis H=6.038; p=0.049); however, post hoc testing revealed no significant difference between any of the three groups for pain improvement (p=0.061 to p=1.000). There was also no significant difference in the magnitude of abduction AROM improvement between the three groups based on the radiographic severity of GHOA (Kruskal-Wallis H=2.887; p=0.236). Finally, there was no significant difference in the magnitude of disability improvement via the Quick DASH between the three groups based on the radiographic severity of GHOA (Kruskal-Wallis H=0.156; p=0.925).

Conclusion

Patients with GHOA referred to PT experience small but statistically significant short-term improvements in pain, abduction AROM, and disability regardless of GHOA radiographic severity. There is no significant association between the magnitude of clinical improvement and the severity of radiographic GHOA. However, despite statistically significant improvements in pain, only patients with mild GHOA experienced clinically significant improvements in pain. Patients with GHOA, regardless of severity, may or may not experience clinically significant improvements in disability after PT.

## Introduction

Glenohumeral osteoarthritis (GHOA) is a common cause of musculoskeletal pain (MSP) that can frequently lead to pain and functional disability in patients throughout the world [1-4]. In addition to clinical symptoms, GHOA is characterized by radiographic joint degeneration, such as loss of cartilage, narrowing of the joint space, and pathological changes in subchondral bone [3,5]. The prevalence of GHOA can be as high as 20% in older individuals (>65 years old), thus indicating significant clinical relevance and the need for effective treatments for this common condition [2,4]. GHOA can be managed with conservative or surgical interventions, although conservative interventions such as physical therapy (PT) are generally first-line interventions depending on the severity of GHOA [3,4].

Despite PT being a common treatment option for GHOA, concrete recommendations for PT for GHOA are currently limited with few studies on the topic [1,3,4]. One recent clinical practice guideline from the American Academy of Orthopedic Surgeons (AAOS) indicates that further research is needed for multiple areas of treatment for GHOA, including PT for GHOA [3]. To date, no study exists that examines the impact of the severity of radiographic GHOA on pain, abduction active range-of-motion (AROM), and functional disability outcomes after referral to conventional PT. The purpose of this retrospective analysis was to examine how conventional PT impacts outcomes for patients with GHOA based on the severity of radiographic GHOA findings to optimize referral practices to outpatient PT to improve patient outcomes.

## Materials and methods

Study design

This study is a retrospective chart review of patients who were referred to PT for MSP and received PT in the outpatient setting between 2016 and 2022 and is aimed at assessing clinical outcomes based on the severity of GHOA via radiograph imaging. This study was approved by the first author’s Institutional Review Board (IRB) under Study #20220636.

Setting, data sources, and participants

Prior to the initiation of this study, feasibility testing was completed by the first author to ensure that enough patients would exist in the hospital system in order to achieve a sufficient sample size. Patient charts were gathered by the University Hospitals Clinical Research Center by searching patients from 2016 to 2022 with ICD-10 code of M19.011 (primary osteoarthritis, right shoulder) or ICD-10 code of M19.012 (primary osteoarthritis, left shoulder), as well as a PT evaluation codes (97161, 97162, 97163). Charts were retrieved from multiple outpatient PT clinics within a single hospital system from 2016 to 2022. After data were de-identified, data were collected and stored on a secure computer server. Patient charts were then searched from the initial PT evaluation to the final PT session for relevant data. A sample size of 220 patients was achieved by practicality after one year of data collection.

Inclusion and exclusion criteria

Patients were included if they received conventional PT in the outpatient setting, received conventional PT for MSP, had shoulder radiograph imaging within two years of initial PT evaluation, had more than one PT visit (i.e. attended a follow-up session after initial evaluation), and did not have a history of shoulder surgery. Patients were excluded if they received PT for diagnoses unrelated to MSP, only received one visit of PT, had a history of shoulder surgery, or did not have shoulder radiograph imaging within two years of initial PT evaluation.

Primary outcomes

The main clinical outcome variables assessed in this study were pain, abduction AROM, and disability. Pain was measured by the visual analog scale (VAS) on a 0-10 point scale. Abduction AROM was assessed via goniometer measurement by the treating clinician and recorded in degrees from 0 to 180 degrees. Disability was assessed via the quick disabilities of the arm, shoulder, and hand (DASH) questionnaire in percentage points on a 0-100 scale, with 0 representing no disability and 100 representing total disability.

Study definitions

For the purposes of this study, patients were stratified into three subgroups depending on the severity of GHOA found in their shoulder radiograph imaging. The “No GHOA” group included patients without any finding of GHOA on radiograph imaging to serve as a control group. The “Mild GHOA” group included patients with mild GHOA on radiograph imaging. The “Moderate/Severe GHOA” group included patients with either moderate or severe GHOA on radiograph imaging. Patients with moderate or severe GHOA were placed into one subgroup due to the limited number of patients in order to allow for proper data analysis. The determination of GHOA severity was based on the written summary provided on the patient's radiograph imaging results in the patient's chart. For the purposes of this study, conventional PT is defined as usual care PT in which the evaluating physical therapist independently chooses the interventions, is not standardized for all patients to mimic "real world" care, and consists of a heterogeneous group of interventions (exercise, manual therapy, and/or physical modalities). 

Data collection

Data collection was performed by multiple authors. The data collected included the number of patients, patient age (years), sex (male/female), laterality of shoulder symptoms (right/left), duration of symptoms prior to the first PT session (days), an impression from radiograph imaging summary, pre- and post-PT pain (VAS, 0-10 scale), pre- and post-PT abduction AROM (degrees), pre- and post-PT disability via the Quick Dash score (0-100 percentage points), and the number of total PT visits.

Statistical analysis

Statistical analysis for this study was performed using the Statistical Package for the Social Sciences (SPSS) version 29.0 (Armonk, NY: IBM Corp). Frequency counts and summative statistics were used to describe the demographics of the included patients. The Kolmogorov-Smirnov test or the Shapiro-Wilk test, depending on sample size, was used to determine the distribution of the data and allow for proper statistical analysis. Comparisons between the three groups were completed using the ANOVA test for parametric data or the Kruskal-Wallis test for nonparametric data. Post-hoc testing was completed using the Bonferroni correction for further analysis of the groups. Subgroup analyses for AROM and disability outcomes were completed using nonparametric tests due to the small sample size. Comparison within groups for pre- and post-PT values was completed using the paired t-test for parametric data and the related-samples Wilcoxon signed-rank test for non-parametric data. Statistical significance was determined to be 0.05 for this study.

## Results

Entire cohort demographics

The entire cohort of included patients (n=220) had a mean age of 62.2±12.4 years, with a mean number of PT visits of 7.8±4.5 visits. A total of 89 included patients (40.5%) were male, and 131 included patients (59.5%) were female. From the included patients (n=220), 114 patients (51.8%) were treated for their right shoulder, and 106 patients (48.2%) were treated for their left shoulder with a mean duration of symptoms of 458.8±1,086.2 days (n=200 patients; 90.9% reported; median=120 days; range=2.0-7,300.0). Refer to Table 1 for information on the entire cohort for this study.

**Table 1:** Demographics of the entire patient cohort (n=220) for the current study. Abbreviations: SD, standard deviation; PT, physical therapy.

Entire cohort demographic information	Values
Patients (n)	220
Mean age ± SD, years	62.2 ± 12.4
Median age (range), years	63.0 (27.0-89.0)
Mean PT visits ± SD	7.8 ± 4.5
Median PT visits (range)	7.0 (2.0-24.0)
Male (n, %)	89 (40.5%)
Female (n, %)	131 (59.5%)
Right shoulder (n, %)	114 (51.8%)
Left shoulder (n, %)	106 (48.2%)
Mean duration of symptoms ± SD, days	458.8 ± 1086.2
Median duration of symptoms (range), days	120.0 (2.0-7,300.0)

Subgroup demographics

Among the subgroups, the No GHOA group (n=104) had a mean age of 56.6±12.6 years, with a mean number of PT visits of 7.5±4.0 visits and a mean duration of symptoms of 418.7±1,219.7 days (n=96; 92.3% reported). The Mild GHOA group (n=61) had a mean age of 68.3±9.6 years, with a mean number of PT visits of 7.5±4.5 and a mean duration of symptoms of 545.4±1,119.7 (n=56; 91.8% reported). The Moderate/Severe GHOA group (n=55) had a mean age of 66.1±10.3 years, with a mean number of PT visits of 8.5±5.2 visits and a mean duration of symptoms of 438.1±713.6 days (n=48; 87.3% reported). There was a significant difference in age between the three groups (F=25.020; p<0.001), with the No GHOA group having a significantly younger cohort than the Mild GHOA and Moderate/Severe groups (p<0.001 and p<0.001, respectively). There was no significant difference in the number of PT visits between the three groups (Kruskal-Wallis H=0.780; p=0.677). Furthermore, there was no significant difference in the duration of symptoms between the three groups (Kruskal-Wallis H=5.461; p=0.065). Refer to Table 2 for more information on subgroup demographics.

**Table 2:** Demographics for the three groups included in this study by the severity of radiographic glenohumeral osteoarthritis (GHOA). Abbreviations: SD, standard deviation; PT, physical therapy; GHOA, glenohumeral osteoarthritis.

Categories	No GHOA group	Mild GHOA group	Moderate/severe GHOA group	Between-group p-values
Total patients (n, %)	104 (47.3%)	61 (27.7%)	55 (25.5%)	-
Mean age ± SD, years	56.6 ± 12.6	68.3 ± 9.6	66.1 ± 10.3	p<0.001
Median age (range), years	58.0 (27.0 - 88.0)	67.0 (41.0 - 89.0)	65.0 (33.0 - 83.0)	
Mean PT visits ± SD, number of visits	7.5 ± 4.0	7.5 ± 4.5	8.5 ± 5.2	p=0.677
Median PT visits (range), number of visits	6.0 (2.0 - 21.0)	7.0 (2.0 - 24.0)	7.0 (2.0 -24.0)	
Mean duration of symptoms ± SD, days	418.7 ± 1,219.7	545.4 ± 1,119.7	438.1 ± 713.6	p=0.065
Median duration of symptoms (range), days	94.0 (2.0 - 7,300.0)	172.0 (11.0 - 4,745.0)	160.0 (6.0 - 4,000.0)	

Pain outcomes

The No GHOA group (n=104), Mild GHOA group (n=61), and Moderate/Severe GHOA group (n=55) each had a significant within-group improvement in pain from pre-PT to post-PT (p<0.001, p<0.001, and p<0.001, respectively). The mean pre-PT pain level was 4.4±2.8 points (median=4.0 points; range=0.0-10.0 points), and the mean post-PT pain level was 3.0±3.0 points (median=2.0 points; range=0.0-10.0 points) for the No GHOA group. The mean pre-PT pain level was 5.0±2.8 points (median=5.0 points; range=0.0-10.0 points), and the mean post-PT pain level was 2.5±2.3 points (median=2.0 points; range-0.0-10.0 points) for the Mild GHOA group. The mean pre-PT pain level was 4.5±3.2 points (median=5.0 points; range=0.0-10.0 points), and the mean post-PT pain level was 3.0±3.0 points (median=3.0 points; range=0.0-10.0 points) for the Moderate/Severe GHOA group (n=55).

There was no significant difference between the pre-PT pain levels between the three groups (Kruskal-Wallis H=1.448; p=0.485). There was initially a significant difference in the magnitude of pain improvement between the three groups based on the radiographic severity of GHOA (Kruskal-Wallis H=6.038; p=0.049); however, post-hoc testing revealed no significant difference between any of the three groups for pain improvement (p=0.061 to p=1.000). The No GHOA group (n=104) had a mean pain improvement of 1.4±2.8 points (median=1.0; range=-7.0-9.0 points), the Mild GHOA group (n=61) had a mean pain improvement of 2.4±2.8 points (median=2.0; range=-3.0-9.0 points), and the Moderate/severe GHOA group (n=55) had a mean pain improvement of 1.5±2.6 points (median=1.0 points; range=-3.0-7.0 points). Refer to Table 3 for information on the improvement of symptoms per subgroup based on the GHOA radiographic severity.

**Table 3:** Improvement in pain, abduction active range-of-motion (AROM), and disability for each of the three groups in this study based on the severity of radiographic glenohumeral osteoarthritis (GHOA). Abbreviations: GHOA, glenohumeral osteoarthritis; AROM, active range-of-motion; DASH, Disability of the arm, shoulder, and hand.

Categories	No GHOA group	Mild GHOA group	Moderate/severe GHOA group	Between-group p-value
Mean pain improvement (points)	1.4 ± 2.8	2.4 ± 2.8	1.5 ± 2.6	p=0.049
Median pain improvement (points)	1.0	2.0	1.0	
Subjects (n,%)	104 (100%)	61 (100%)	55 (100%)	
Mean abduction AROM improvement (degrees)	15.2 ± 27.5	8.3 ± 16.7	19.8 ± 22.7	p=0.236
Median abduction AROM improvement (degrees)	10.0	5.0	15.0	
Subjects (n,%)	44 (42.3%)	21 (34.4%)	29 (52.7%)	
Mean Quick DASH disability score improvement (%)	14.3 ± 21.0	16.3 ± 19.8	14.0 ± 13.7	p=0.925
Median Quick DASH disability score improvement (%)	11.5	12.0	12.0	
Subjects (n, %)	35	23	18	

AROM outcomes

The No GHOA group (n=44), the Mild GHOA group (n=44), and the Moderate/Severe GHOA group (n=29) each had a significant within-group improvement in abduction AROM from pre-PT to post-PT (p<0.001, p=0.011, and p<0.001, respectively). The mean pre-PT abduction AROM was 103.1±42.2 degrees (median=95.0 degrees; range=10.0-180.0 degrees), and the mean post-PT abduction AROM was 118.3±38.7 degrees (median=119.5 degrees; range=30.0-180.0 degrees) for the No GHOA group (n=44). The mean pre-PT abduction AROM was 123.9±39.1 degrees (median=135.0 degrees; range=30.0-180.0 degrees), and the mean post-PT abduction AROM was 132.1±38.8 degrees (median=140.0 degrees; range=45.0-175.0 degrees) for the Mild GHOA group (n=21). The pre-PT abduction AROM was 91.6±29.9 degrees (median=94.0 degrees; range=45.0-166.0 degrees), and the post-PT abduction AROM was 111.4±33.3 degrees (median=114.0 degrees; range=46.0-175.0 degrees).

For subgroup analysis of all patients with pre- and post-PT AROM (n=94), there was a significant difference in pre-PT abduction AROM between the three groups (Kruskal-Wallis H=8.274; p=0.016) with post-hoc testing indicating a significance difference between the Mild GHOA and Moderate/Severe GHOA groups (p=0.013). However, there was no significant difference in the magnitude of abduction AROM improvement between the three groups based on the radiographic severity of GHOA (Kruskal-Wallis H=2.887; p=0.236). The No GHOA group (n=44) had a mean abduction AROM improvement of 15.2 ± 27.5 degrees (median=10.0 degrees; range=-57.0-75.0 degrees), the Mild GHOA group (n=21) had a mean abduction AROM improvement of 8.3±16.7 degrees (median=5.0 degrees; range=-27.0-60.0 degrees), and the Moderate/Severe GHOA group (n=29) had a mean abduction AROM improvement of 19.8±22.7 degrees (median=15.0 degrees; range=-7.0-85.0 degrees). Refer to Table 3 for information on the improvement of symptoms per subgroup based on the GHOA radiographic severity.

Disability outcomes

The No GHOA group (n=35), the Mild GHOA group (n=23), and the Moderate/Severe GHOA group (n=18) each had a significant within-group improvement in disability from pre-PT to post-PT (p<0.001, p<0.001, and p<0.001, respectively). The mean pre-PT Quick DASH score was 51.1±22.5 percentage points (median=47.0 percentage points; range=6.8-95.5 percentage points), and the mean post-PT Quick DASH score was 36.8±22.9 percentage points (median=38.0 percentage points; range=0.0-95.0 percentage points). The mean pre-PT Quick DASH score was 38.5±24.7 percentage points (median=38.6 percentage points; range=0.0-95.0 percentage points), and the mean post-PT Quick DASH score was 22.2±16.8 percentage points (median=22.0 percentage points; range=0.0-54.55% percentage points) for the Mild GHOA group (n=23). The mean pre-PT Quick DASH score was 46.9±18.7 percentage points (median=49.0 percentage points; range=9.1-73.0 percentage points), and the mean post-PT Quick DASH score was 32.9±17.8 percentage points (median=27.0 percentage points; range=6.0-61.0 percentage points).

For subgroup analysis of all patients with pre- and post-PT Quick DASH scores (n=76), there was no significant difference in pre-PT Quick DASH scores between the three groups (Kruskal-Wallis H=4.073; p=0.130). There was no significant difference in the magnitude of disability improvement via the Quick DASH between the three groups based on the radiographic severity of GHOA (Kruskal-Wallis H=0.156; p=0.925). The No GHOA group (n=35) had a mean improvement in disability of 14.3±21.0 percentage points (median=11.5 percentage points; range=-21.0-57.5 percentage points), the Mild GHOA group (n=23) had a mean improvement in disability of 16.3±19.8 percentage points (median=12.0 percentage points; range=-18.0-59.0 percentage points), and the Moderate/Severe GHOA group (n=18) had a mean improvement in disability of 14.0±13.7 percentage points (median=12.0 percentage points; range=-5.1-48.0 percentage points). Refer to Table 3 for information on the improvement of symptoms per subgroup based on the GHOA radiographic severity.

## Discussion

This study represents the first retrospective analysis to date to examine the impact of PT on patient outcomes based on the radiographic severity of GHOA. Although GHOA can be managed both conservatively and surgically, a great deal of attention in the literature has been given to surgical interventions, while relatively little exists on PT interventions [6-9]. Furthermore, this study assists the clinician in deciding which patients suffering from shoulder-related MSP may be most appropriate for PT referral if GHOA is involved and surgical intervention is not warranted [1,3-5].

Based on the results of this study, PT may result in small but statistically significant improvements in pain, disability, and function, irrespective of radiographically determined GHOA severity. Based on a minimal clinically important difference (MCID) of 1.4/10 points as reported elsewhere in the literature for MSP, the median was 1.0 points for the No GHOA group, 2.0 points for the Mild GHOA group, and 1.0 points for the Moderate/Severe group [10,11]. Therefore, only the Mild GHOA group had clinically significant improvements in pain after PT, and the small improvements in the No GHOA and Moderate/Severe groups did not reach clinical significance. This is consistent with other studies in the literature examining other causes of MSP, such as rotator cuff disease, in which PT interventions may or may not lead to clinically significant improvements in pain [12,13]. There was also notable variability in the magnitude of pain improvement in each of the subgroups, indicating that other factors could be playing a large role in the change of symptoms throughout the patient’s time at PT. One potential reason for the lack of clinical significance in the No GHOA group is that these patients were likely sent to PT for other causes of MSP that were not controlled by this study, such as rotator cuff pathology or adhesive capsulitis, since these patients did not have GHOA [4,9,14,15]. However, it is also possible that patients in the Mild GHOA and Moderate/Severe GHOA groups also had additional shoulder pathologies that could confound these results, thus limiting the application of this study. 

For abduction AROM outcomes, there was also significant variability in the change in AROM after PT in the No GHOA group (-57.0 to 75 degrees), the Mild GHOA group (-27.0 to 60.0 degrees), and the Moderate/Severe GHOA group (-7.0 to 85.0 degrees). Interestingly, the Mild GHOA group had the lowest median improvement in abduction AROM of 5.0 degrees as compared to the improvements in abduction AROM of 10.0 and 15.0 degrees in the No GHOA and Moderate/Severe groups, respectively. The MCID for disability via the Quick DASH ranges from 8/100 points to 20/100 points, although this is debated in the literature [16,17]. When using the lower threshold, all groups had a clinically significant improvement in median disability of 11.5 percentage points, 12.0 percentage points, and 12.0 percentage points in the No GHOA, Mild GHOA, and Moderate/Severe GHOA groups, respectively. As with the other clinical outcomes, there was great variability in the magnitude of disability improvement in each group. However, if the upper threshold for MCID for the Quick DASH is used, none of the three groups experienced clinically significant improvements in disability. Therefore, the question remains as to the clinical impact of unstandardized conventional PT on disability related to GHOA, regardless of severity. 

It is also important to note that all three groups had similar levels of PT throughout the study, with a median of 7.0 visits for the entire cohort. It is unknown if the number of PT visits may affect clinical outcomes, as this study could present patients who were treated with too few visits to see clinically significant changes. Because of a small number of visits, the results of this study only indicate short-term results, and more research is needed to determine how increased visits impact both short- and long-term results. Overall, the results of this study cautiously suggest that patients with GHOA may experience large variability in pain, AROM, and disability outcomes with referral to PT. Clinicians can use this clinical outcome information when deciding if PT referral is appropriate for patients with GHOA while also considering other relevant factors, such as cost, possibility of future surgery, and patient preferences.

There are several limitations that impact the generalization and application of these study results. One such limitation is the nature of a retrospective analysis and the fact that subjects were not controlled for various biases, such as selection bias. It is highly likely that patients with differing severity of radiographic GHOA are referred to PT at differing rates, potentially complicating results that can be drawn from this study. Future randomized controlled trials are needed to control for these biases to determine if PT does, in fact, provide significant comparable improvements in outcomes based on the radiographic severity. Furthermore, the radiographic severity of GHOA was determined via radiograph results listed in the patient’s radiograph report and determined by the first author, which can result in misclassification bias as an ideal study would utilize classification by direct radiograph reading from a specialist orthopedic surgeon or radiologist. GHOA can have varying symptoms, and the literature indicates that the deformity present in GHOA can be complex and not easily classified, thus increasing the possibility of grouping together patients that are very different in terms of the burden of GHOA [2,3,18]. Another limitation centers on the fact that the three groups were not equal in terms of patient demographics; the No GHOA group, for instance, was significantly younger than the Mild GHOA and Moderate/Severe GHOA groups. This observation may just be a natural consequence of this comparison as GHOA is more common in older individuals [5,19]. It is important to note that the number of PT visits per group and the duration of symptoms for each group were not significantly different, thus improving confidence in the comparisons drawn in this study. However, future studies should examine outcomes based on the severity of GHOA with patient groups that are equal across all demographic fields. Likewise, there was a wide variation in the number of visits given within each group, likely skewing the results. 

Another limitation is the fact that interventions for conventional PT for each group were not controlled. While this was done to mimic the real-world effectiveness of PT for GHOA, it is highly possible that different groups may have received different interventions during PT, as various PT interventions exist for the management of GHOA [20-22]. Finally, this study had a relatively small sample size. It is possible that a larger sample size would demonstrate that lower levels of GHOA are associated with greater outcomes after PT, although this concept remains to be seen as this study did not find a significant difference in the magnitude of outcome improvement based on GHOA severity. However, it is important to note that all groups, regardless of GHOA radiographic severity, experienced small but statistically significant improvements in pain and disability after PT, although several of these improvements did not reach clinical significance. Despite this observation, this study did not contain a group that did not receive PT as a control group, masking the impact that PT had on outcomes as compared to simple improvement with time. Overall, further high-level evidence is needed to examine the impact and utilization of PT based on the GHOA radiographic severity in order to decrease cost, optimize referral patterns, and improve patient outcomes.

## Conclusions

Patients with GHOA referred to PT experience small but statistically significant short-term improvements in pain, abduction AROM, and disability regardless of the GHOA radiographic severity. There is no significant association between the magnitude of clinical improvement and the severity of radiographic GHOA. However, despite statistically significant improvements in pain, only patients with mild GHOA experienced clinically significant improvements in pain. Patients with GHOA, regardless of severity, may or may not experience clinically significant improvements in disability after PT. In an attempt to mimic real-world effectiveness, this study did not control for specific PT interventions, and thus more research is needed to determine how variations in PT quantity, quality, or type impact outcomes in patients with GHOA.
